# Effects of an extracted lectin from *Citrullus colocynthis* L. (Cucurbitaceae) on survival, digestion and energy reserves of *Ectomyelois ceratoniae* Zeller (Lepidoptera: Pyralidae)

**DOI:** 10.3389/fphys.2013.00328

**Published:** 2013-11-12

**Authors:** Samar Ramzi, Ahad Sahragard, Jalal J. Sendi, Ali Aalami

**Affiliations:** ^1^Department of Plant Protection, Faculty of Agricultural Science, University of GuilanRasht, Iran; ^2^Department of Agronomy and Plant Breeding, Faculty of Agricultural Science, University of GuilanRasht, Iran

**Keywords:** lectin, *Citrullus colocynthis*, *Ectomyelois ceratoniae*, larval survival, digestive physiology

## Abstract

Lectins are the heterogeneous proteins in plants that serve as storage proteins via defensive mechanisms against herbivores. In the current study, a lectin was extracted and purified from seeds of *Citrullus colocynthis* by Sepharose 4B-Galactose and DEAE-cellulose fast flow chromatographies. Different concentrations of the lectin were added to artificial diet of *Ectomyelois ceratoniae* larvae finding out its effect on some biological parameters, digestive physiology and amount of storage macromolecules. It was found that CCA (*C. colocynthis* Agglutinin) increased life span from 23.44 days in control to 28.59 days in the treated individuals. Survival of larvae on control and CCA diets were 93.3 and 66.6%, respectively. Different concentrations of CCA significantly affected α-amylase and general proteolytic activities except for TAG-lipase activity. Activities of all specific proteases decreased when larvae were fed on different concentrations of CCA except for aminopeptidase. Meanwhile, amount of storage macromolecules in the larvae fed on different concentrations of CCA statistically decreased vs. control. These results demonstrated that CCA could intervene in physiology of *E. ceratoniae* and survival of larvae. Therefore, it can be taken into consideration in IPM of the pest through plant breeding programs.

## Introduction

When an insect feeds on an host plant, it may ingest some proteins by toxic effects (Ffrench-Constant and Bowen, 2000; Carlini and Grossi-de-Sá, 2002; Sanchis and Bourguet, 2008). These proteins could be divided into several groups like proteinase inhibitors, botanical compounds (Toxic materials extracted from plants), ribosome inactivating proteins and plant lectins (Gatehouse et al., 1995; Czapla, 1997; Vasconcelos and Oliveira, 2004; Vandenborre et al., 2009). Lectins are a group of heterogeneous proteins that bind reversibly to specific mono or oligosaccharides (Peumans and Van Damme, 1995). These proteins have been extracted from several organisms like plants, fungi, bacteria and vertebrates that are involved in different physiological processes. In plants, they have a crucial role in defense against pathogens and herbivores so that they have been evolved as a preferentially binding agents to foreign glycans. Different studies have shown reduced performance of several insect species after adding of lectins to their diets that may confirm lectins as storage proteins to be used against herbivores (Peumans and Van Damme, 1995; Rahbé et al., 1995; Sauvion et al., 2004; Michiels et al., 2010; Shahidi-Noghabi et al., 2010). Various entomotoxic lectins have been extracted from plants like lectins ASA I and ASA II from *Allium sativum*, rice, legumes, and cucurbitaceae (Peumans and Van Damme, 1995; Zhu-Salzmana et al., 2002; Jiang et al., 2006; Van Damme et al., 2008; Clement and Venkatesh, 2010; Clement et al., 2010; Marzouk et al., 2012).

Bitter apple or *Citrullus colocynthis* L. (Cucurbitaceae) is a medicinal plant in Africa and Asia (Tavakkol-Afshari et al., 2005). Fruits of the plant contain bitter glycoside and those are used as drug for several purposes. Compounds in the fruits have anti-inflammatory properties due to presence of alkaloids and flavonoids so those are used to treat pain and rheumatoid arthritis (Marzouk et al., 2012). Meanwhile, aqueous and methanolic extracts of the plant demonstrated high anti-microbial activity against some bacteria and fungi. Several studies have also shown anti-cancer and apoptosis properties of seeds as well as their role to decrease blood sugar (Tannin-Spitz et al., 2007).

*Ectomyelois ceratoniae* Zeller (Lepidoptera: Pyralidae) is the major pest of pomegranate and many stored products that causes 15–90% of damages, annually. Adults lay their eggs on pomegranate crown, larvae then hatch and feed on tissues around pomegranate grains (Farazmand et al., 2008). Larvae hibernate in infested fruits on soil surface. Although different control methods were used such as collecting the infested fruits, removal of pomegranate crown, release of biocontrol agents but no effective control has yet been achieved (Farazmand et al., 2008). Since, larvae fed on inner parts of the fruits, insecticides could not reach the larvae so chemical spraying is not effective. Therefore, using plant inhibitors to provide resistant varieties may be promising to decrease population outbreaks and damages caused by *E. ceratoniae*. Since lectins are exposed to midgut they could interact by epithelial cells and induce apoptotic processes. For example, Hamshou et al. (2010) demonstrated that the *Sclerotinia sclerotiorum* lectin is clearly targeting the insect midgut. In our previous study, it was obtained negative effects of CCA (*C. colocynthis* Agglutinin) on amylolytic activity of *E. ceratoniae in vitro* (Ramzi and Sahragard, in press). Hence, a comprehensive understanding of CCA interactions with the insects is mandatory before using these plant materials. So, the objectives of this study are to find out the possible effects of CCA on survival, pupal weight, digestive physiology, and storage macromolecules of *E. ceratoniae* larvae.

## Materials and methods

### Insect rearing

Larvae of *E. ceratoniae* were collected from pomegranate orchards and reared on artificial diet containing wheat bran (100 g), yeast (3 g), sugar (10 g), glycerine (40 ml), and water (40 ml) for at least five generations to have a homogeneous stock population at 28 ± 2°C, 85% of RH and 16:8 h. L:D (Zare, in press).

### Preparation of sepharose4B-galactose column

In order to prepare the column, 20 ml of Sepharose 4B was suspended in 40 ml of 0.5 M Na_2_CO_2_ (pH 11.0). Two ml divinylsulphone were added to the suspension then, the mixture was incubated for 70 min at room temperature with gentle shaking. After activation, 500 mg of galactose in 50 ml 0.5 M Na_2_CO_2_ (pH 11.0) was added and the suspension was re-incubated at room temperature for 12 h with gentle shaking. The sorbent was washed with water; the unbound arm was blocked with b-mercaptoethanol-containing buffer, and then packed into the column. The sorbent was equilibrated with Tris-HCl 0.1 M and was used for the affinity purification of CCA (Bulgakova et al., 2004).

### Purification of CCA

Seeds of *C. colocynthis* were grounded to be a fine powder using a mill device. The dry powder was incubated in phosphate buffer (0.1 M pH 7.1) for approximately 20 h at 4°C. The mixture was then centrifuged at 5000 rpm for 20 min and remaining debris removed by passing the supernatant through filter paper (Whatmann No.4) (Hamshou et al., 2010). Supernatant was precipitated by 0–60% concentrations of ammonium sulfate and centrifuged at 5000 rpm for 20 min. Debris was eluted in Tris-HCl buffer (0.1 M, pH 7) and dialyzed in the same buffer overnight (de Oliveira et al., 2011). Affinity chromatography was performed on a Sepharose 4B-galactose column equilibrated with Tris-HCl buffer (0.1 M, pH 7). After loading the extract, the affinity column was washed with buffer and the lectin bound to the column was eluted with 20 mM 1,3-diaminopropane (DAP) (Hamshou et al., 2010). Fractions showing the highest protein content were pooled and used for forthcoming steps. Obtained fractions after the first affinity chromatography were loaded on an anion exchange chromatography column of DEAE-Cellulose fast flow, equilibrated with DAP (Hamshou et al., 2010). After washing with DAP the lectin was eluted using Tris–HCl (0.1 M, pH 7.0) containing 0.5 M NaCl. Finally, the lectin fractions were dialyzed against water and lyophilized. The purity of the lectin was analyzed by SDS-polyacrylamide gel electrophoresis (PAGE).

### Effect of CCA on survival, life duration, and pupal weight

Artificial diets containing 0 (as control), 0.5, 1, and 2% of CCA were prepared and 60 newly laid eggs of *E. ceratoniae* were put on each diet. Hatched larvae were allowed to feed on control and treated diets to adult emergence. Development time from 1st larval instar to adult, larval survivorship and pupal weight were recorded and compared with control.

### Effect of CCA on digestive enzyme activities and storage macromolecules

Different concentrations of lectin (0, 0.5, 1, and 2%) were prepared and 1st instar larvae of *E. ceratoniae* were allowed to feed on the diets containing CCA (*N* = 30 in each concentration with three replicates). When the larvae reached to first half of 5th larval instars, they were dissected and their midgut and fat bodies were used to measure biochemical parameters including digestive enzymes and storage macromolecules.

### Sample preparation

Fifth larval instars were dissected and the midgut appeared after removal of fat bodies and other organs, rinsed in ice-cold distilled water, placed in a pre-cooled homogenizer and grounded before centrifugation. Equal portions of larval midgut and distilled water were used to have a desirable concentration of the enzymes (W/V). Homogenates were separately transferred to 1.5 ml centrifuge tubes and centrifuged at 13,000 rpm for 20 min at 4°C. The supernatants were pooled and stored at −20°C for subsequent analyses.

### α-Amylase assay

The method described by Bernfeld (1955) was used to assay α-amylase activity. Ten microlitres of the enzyme were incubated for 30 min at 35°C with 50 μl of phosphate buffer (0.02 M, pH 7.1) and 20 μl of soluble starch (1%) as substrate. The reaction was stopped by addition of 80 μl dinitrosalicylic acid (DNS) and heating in boiling water for 10 min prior to read absorbance at 545 nm. One unit of α-amylase activity was defined as the amount of enzyme required to produce 1 mg maltose in 30 min at 35°C. The negative control contained all reaction mixtures with pre-boiled enzyme (for 15 min) to prove the enzyme presence in the samples.

### TAG-lipase assay

The enzyme assay was carried out as described by Tsujita et al. (1989). Twenty μl of gut extract and 40 μl of *p-nitrophenyl* butyrate (27 mM) as substrate were incorporated by 100 μl of universal buffer (10 mM, pH 11), mixed thoroughly and incubated at 37°C. After 1 min, 100 μl of NaoH (1 M) were added to each tube (control and treatment) and absorbance was read at 405 nm. One unit of enzyme will release 1.0 nmol of *p-nitrophenol* per min at pH 7.2 and 37°C when *p*-nitrophenyl butyrate was used as substrate. Standard curve of *p*-nitrophenol was used to calculate the specific activity of the enzyme.

### General proteases assay

Cohen's (1993) method was used to assay the general proteolytic activity in the midgut of *E. ceratoniae* fed on different concentrations of CCA by using hemoglobin (20 mg/ml) as substrate (Cohen, 1993). Hemoglobin solution (50 μl) was added to 100 μl of universal buffer solution (pH 9) and incubation at 30°C was initiated after addition of 20 μl of enzyme solution for 120 min. For termination of proteolysis, 150 μl of 10% TCA was added to the reaction mixture; precipitation was achieved by cooling at 4°C for 45 min and the reaction mixture was centrifuged at 13000 rpm for 10 min. Blank solution contained all mentioned portions except for enzyme. The peptides liberated from hemoglobin were estimated using Folin-phenol reagent at 630 nm (Folin and Ciocalteu, 1927).

### Determination of specific proteases presence

#### Serine proteinases

Trypsin-, chymotrypsin- and elastase-like activities (as three subclasses of serine proteinases) were assayed using a concentration 1 mM of BApNA (Nabenzoyl-L-arginine-p-nitroanilide), 1 mM SAAPPpNA (N-succinyl-alanine-alanine-proline-phenylalanine-p-nitroanilide), and 1 mM SAAApNA (N-succinyl-alanine-alanine-alanine-p-nitroanilide) as substrates, respectively. The reaction mixture included 35 μl of Tris-HCl buffer (20 mM, pH 8 as literature recommended pH for saerines), 5 μl of each mentioned substrate and 5 μl of enzyme solution. The reaction mixture was incubated at 30°C for a period from 0 to 10 min before adding 30% TCA to terminate the reaction. The absorbance of the resulting mixture was then measured spectrophotometrically at 405 nm by *p*-nitroaniline release. To prove the specific proteolytic activity, a negative control were provided for each substrate separately containing all mentioned components except for enzyme pre-boiled at 100°C for 30 min (Oppert et al., 1997).

#### Cysteine proteinases

Cathepsin B, L and D activities (as three subclasses of cysteine proteinases) were assayed using a concentration 1mM of Z-Ala-Arg-Arg 4-metjoxy-β-naphtylamide acetate, N-Benzoyl-Phe-Val-Arg-p-nitroanilide hydrochloride, and cathepsin D (Sigma-Aldricht Co. Switzerland, LM0342) as substrates, respectively. The reaction mixture consisted 35 μl of Tris-HCl buffer (pH 5 as literature recommended pH for cysteines), 5 μl of each mentioned substrate and 5 μl of enzyme solution. The reaction mixture was incubated at 30°C for a period from 0 to 10 min before adding 30% TCA to terminate the reaction and read at 405 nm. To prove the specific proteolytic activity, a negative control were provided for each substrate separately containing all mentioned components except for enzyme pre-boiled at 100°C for 30 min (Oppert et al., 1997).

#### Exopeptidases

Activities of the two exopeptidase in the midgut of *E. ceratoniae* were obtained using Hippuryl-L-Arginine and Hippuryl-L-Phenilalanine for amino- and carboxypeptidases, respectively. The reaction mixture consisted 35 μl of Tris-HCl buffer (pH 7 as literature recommended pH for exopeptidases), 5 μl of each mentioned substrate and 5 μl of enzyme solution. The reaction mixture was incubated at 30°C for a period from 0 to 10 min before adding 30% TCA to terminate the reaction and read at 340 nm. To prove the specific proteolytic activity, a negative control were provided for each substrate separately containing all mentioned components except for enzyme pre-boiled at 100°C for 30 min (Oppert et al., 1997).

### Effect of CCA feeding on energy reservoirs of *E. ceratoniae* larvae

#### Protein determination

Concentration of soluble protein in the fat bodies of 30 larvae of *E. ceratoniae* fed on different concentrations of CCL from 5th larval instars was measured according to the method of Lowry et al. (1951) using bovine serum albumin as standard. Meanwhile, samples of each treatment was unified as the amount of protein.

#### Triacylglyceride determination

A diagnostic kit from PARS-AZMOON® Co. was used to measure the amount of triacylglyceride in the fourth instar larvaeof *E. ceratoniae*. Reagent solution contained phosphate buffer (50 mM, pH 7.2), 4-chlorophenol (4 mM), Adnosine Triphosphate (2 mM), Mg^2+^ (15 mM), glycerokinase 0.4 kU/L), peroxidase (2 kU/L), lipoprotein lipase (2 kU/L), 4-aminoantipyrine (0.5 mM), and glycerol-3-phosphate-oxidase (0.5 kU/L). Samples (10 μl) were incubated with 10 μl distilled water and 70 μl of reagent for 20 min at 25°C (Fossati and Prencipe, 1982). ODs of samples and reagent as standard were read at 546 nm. Following equation was used to calculate the amount of triacylglyceride:

$$\frac{\text{mg}}{\text{dl}}=\frac{\text{OD of sample}}{\text{OD of standard}}\times 0.01126$$

#### Glycogen determination

Fat bodies of 30 larvae were cut and immersed in 1 ml of 30% KOH w/Na_2_SO_4_. Tubes containing samples were covered with foil (to avoid evaporation) and boiled for 20–30 min until complete. Tubes were shaked and cooled in ice. Then, 2 ml of 95% EtOH was added to precipitate glycogen from digested solution. Samples were again shaked and incubated in ice for 30 min. Tubes were centrifuged 13000 rpm for 30 min. Supernatant was removed and pellets (glycogen) were re-dissolved in 1 ml of distilled water before being shaked. Glycogen standard (0, 25, 50, 75, and 100 mg/ml) was prepared before adding phenol 5%. Incubation was performed on ice bath for 30min. Standards and samples were read at 490 nm and distilled water was used as blank (Chun and Yin, 1988).

### Zymogram analyses of digestive enzymes

Zymogram analyses were carried out finding the effect of different concentrations of CCA on digestive enzymes. The activities of enzymes were performed by non-denaturing PAGE. Native-PAGE was performed in a 10% (w/v) separating gel and a 4% stacking gel. The electrode buffer was prepared based on the method of Laemmli (1970) but SDS was not used. The sample buffer contained 25% stacking buffer (0.5 M/L Tris–HCl [pH 6.8]), 20% glycerol, 0.005% (w/v) bromophenol blue, but no mercaptoethanol was added and it was not heated. ^36^ Electrophoresis was conducted at room temperature and 100 V until the blue dye reached the bottom of the slab gel.

#### A-amylase zymogram

The method described by Campos et al. (1990) was used to visualize the amylolytic activity in samples. To gel preparation for α-amylase assay, the gel was rinsed with distilled water and washed by shaking gently with 1% (v/v) Triton X-100 in phosphate buffer containing 2 mmol/l CaCl_2_ and 10 mmol/l NaCl for 1.5 h. The gel was then rinsed with distilled water and treated with a solution of 1.3% I 2, 3% KI to stop the reaction and to stain the unreacted starch background. Zones of α-amylase activity appeared as light bands against a dark background (Campos et al., 1990).

#### TAG-lipase zymogram

Zymogram analysis of lipase was carried out using 12% resolving and 4% stacking gel. After loading of the samples, gel was run at 4°C and constant voltage of 100 mV. The gel was gently separated from glasses and immediately immersed in 5 mM of MU-butyrate solution as fluorescent substrate. After 30 min, gel was put on a UV trans-illuminator to observe white bands in dark background (Zibaee et al., 2011).

#### Proteolytic zymogram

Electrophoretic detection of proteolytic enzymes was performed according to the method described by Garcia-Carreno et al. (1993) Non-reducing PAGE was carried out at 4°C by using gels containing 0.5% gelatin. When dye reached at the end of glass, the gel was gently removed, washed with distilled water and immediately fixed and stained with 0.1% Coomassie brilliant blue R-250 in methanol–acetic acid–water (50:10:40) overnight. Destaining was done in methanol–acetic acid– water (50:10:40).

### Statistical analysis

All data obtained from a complete randomized design were compared by one-way analysis of variance (ANOVA) followed by Tukey's test when significant differences were found at *P* ≤ 0.05. Differences between samplings were considered statistically significant at a probability less than 5% and marked in figures and tables.

## Results

**Figure 1:**
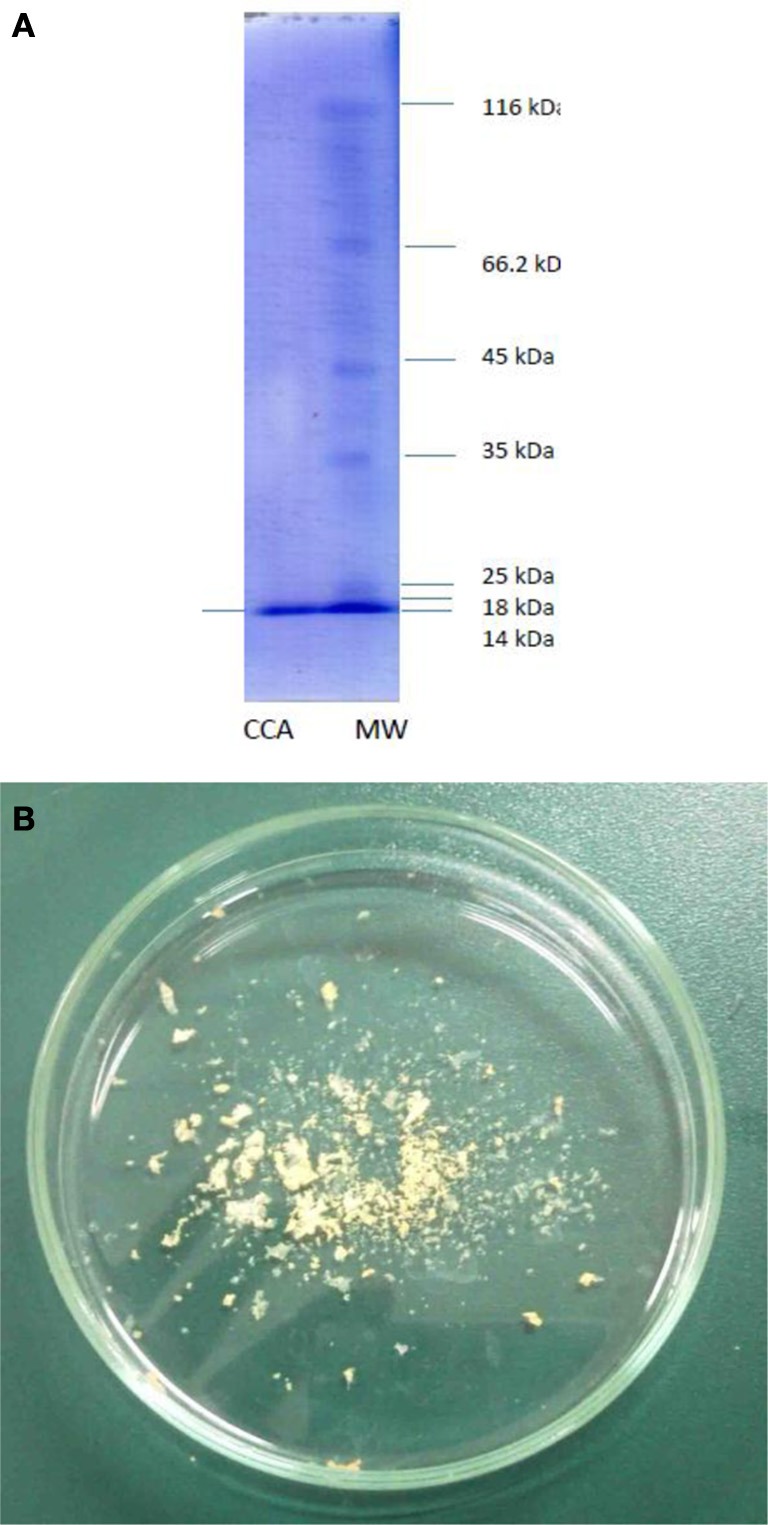
**Purified CCA after column chromatography procedures**. **(A)** Molecular weight. **(B)** Freezdried protein.

Purification process of the crude sample from *C. colocynthis* revealed a purified protein by molecular weight of 14.4 kDa (Figures 1). Involvement of CCA into artificial diet of *E. ceratoniae* caused statistical changes in life cycle, survival and pupal weight of the pest vs. control (Table 1). The pre-adult period increased to 28.59 days on larvae fed on the diet containing 2% CCA in comparison with control by reducing of survival to 66.6% (Table 1). Larval Feeding on different concentrations of CCA revealed a statistical reduction and abnormality in the pupal weight and morphology (Table 1).

**Table 1:** **Pre-adult period, survival, and pupal weight of *E. ceratoniae* fed on different concentration of CCA**.

	**Control**	**0.5%**	**1%**	**2%**
Life cylce^1^ (day)	23.44 ± 0.06a	23.26 ± 0.7a	28.52 ± 0.35b	28.59 ± 0.22c
Survival (%)	93.3 ± 8.23a	93.3 ± 4.19a	83.2 ± 3.17b	66.6 ± 2.87c
Pupal weight (mg)	45.33 ± 0.72a	44.33 ± 0.98a	43 ± 3.09ab	33 ± 2.94b

1 From 1st larval instar to adult emergence.

Values showing statistical differences are shown in various letters in each row.

**Figure 2:**
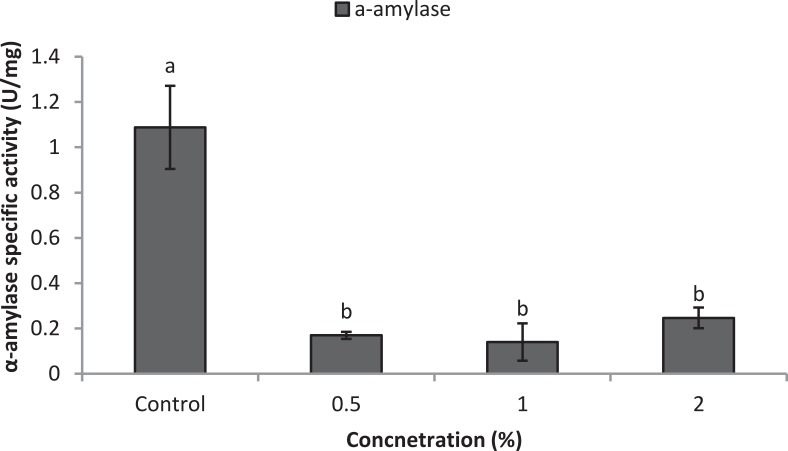
**Effect of CCA on α-amylase activity in the treated larvae of *E. ceratoniae***. Different Letters show statistical differences (Tukey's test, *p* ≤ 0.05).

**Figure 3:**
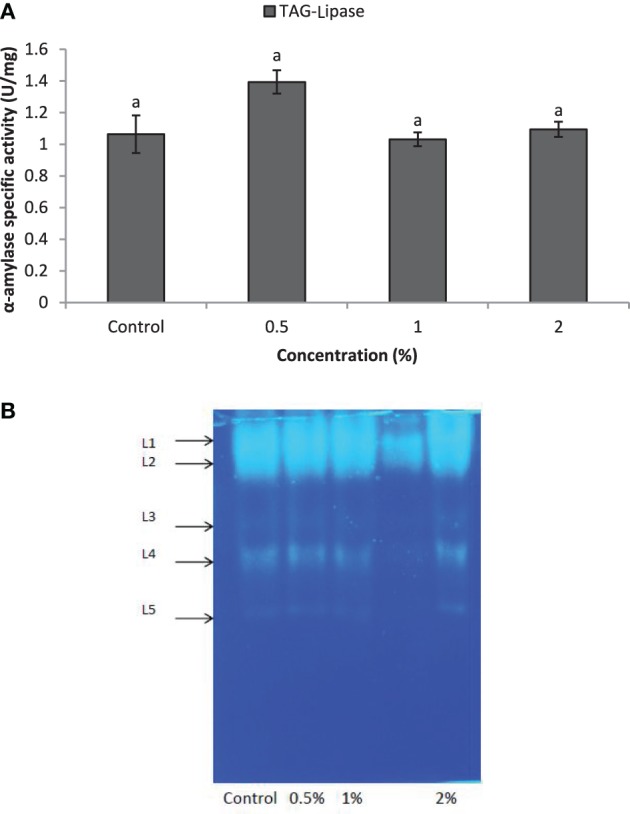
**Effect of CCA on TAG-lipase activity in the treated larvae of *E. ceratoniae***. **(A)** Biochemical assay. **(B)** Native-PAGE electrophoresis. Different Letters show statistical differences of values (Tukey's test, *p* ≤ 0.05).

**Figure 4:**
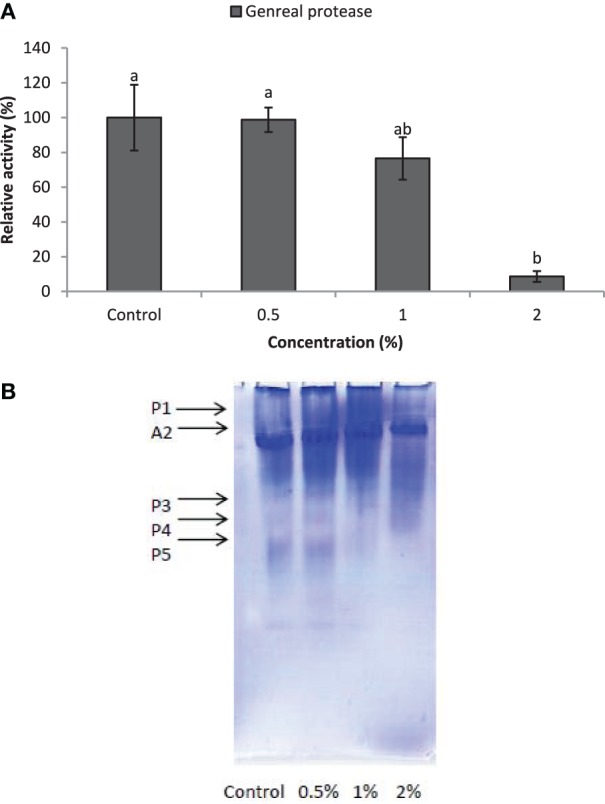
**Effect of CCA on general proteolytic activity in the treated larvae of *E. ceratoniae***. **(A)** Biochemical assay. **(B)** Native-PAGE electrophoresis. Different Letters show statistical differences of values (Tukey's test, *p* ≤ 0.05).

Larvae of *E. ceratoniae* were allowed to feed on the diet containing 0, 0.5, 1, and 2% of CCA from 1st to the first half of 5th larval instar to find out any changes in digestive enzyme activities. The α-amylase activity decreased statistically in the larvae fed on different concentrations of CCA (Figure 2). No statistical differences was observed regarding TAG-lipase activity in treated and control larvae in both enzymatic assay and gel electrophoresis (Figures 3A,B). In case of general proteolytic activity, no statistical differences was observed between control and larvae fed on CCA 0.5% but proteolytic activity in the larvae fed on CCA 2% sharply decreased and showed significant differences (Figure 4A). In gel electrophoresis, five proteolytic bands were observed in control that P3–P5 disappeared or their sharpness decreased in the larvae fed on CCA 2% (Figure 4B).

Since digestive proteases in insects are divided into several classes by different roles in protein digestions, activities of specific digestive proteases in the midgut of *E. ceratoniae* larvae were assayed in control and treated larvae by CCA. Activities of trypsin-like, chymotrypsin-like and elastase-like proteases as three subclasses of serines statistically decreased in the larvae fed on different concentrations of CCA (Table 2). In case of exopeptidases, no statistical difference was observed in case of aminopeptidase activity but activity of carboxypeptidase decreased in the larvae fed on diet containing 1 and 2% of CCA (Table 2).

**Table 2:** **Activity changes of digestive proteases (U/mg protein) after feeding of *E. ceratoniae* larvae on different concentrations of CCA**.

**Specific protease**	**Control**	**0.5%**	**1%**	**2%**
Trypsin-like	2.56 ± 0.12a	1.72 ± 0.24b	1.11 ± 0.14b	1.37 ± 0.14b
Chymotrypsin-like	4.45 ± 0.039a	3.49 ± 0.38b	2.55 ± 0.064c	2.57 ± 0.19c
Elastas-like	2.18 ± 0.072a	0.92 ± 0.095b	1.024 ± 0.085b	0.88 ± 0.047b
Aminopeptidase	4.55 ± 0.41a	5.02 ± 0.32a	4.51 ± 0.44a	4.78 ± 0.33a
Carboxypeptidase	8.45 ± 1.39a	4.20 ± 0.45ab	3.21 ± 0.74b	2.61 ± 0.096b

At each row, different letters show statistical differences of values (Tukey's test, p ≤ 0.05).

**Figure 5:**
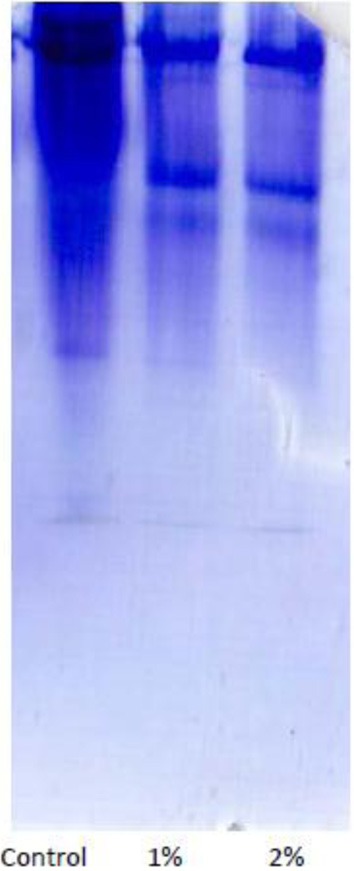
**Protein pattern in fat bodies of the *E. ceratoniae* larvae fed on diets containing 0–2% of CCA**.

Amounts of protein, glycogen and triacylglyceride were evaluated to find possible effects of CCA on storage macromolecules of 5th larval instars. Their amounts decreased statistically in the larvae fed on different concentrations of CCA except for glycogen that no statistical differences was observed between control and larvae treated by CCA 0.5% (Table 3). Also, protein pattern in fat bodies was shown by SDS-PAGE electrophoresis showing significant decrease in number and intensity of bands in the larvae fed on CCA 1 and 2% (Figure 5).

**Table 3:** **Changes of storage macromolecules (mg/dl) after feeding of *E. ceratoniae* larvae on different concentrations of CCA**.

**Storage macromolecules**	**Control**	**0.5%**	**1%**	**2%**
Protein	1.32 ± 0.65a	0.94 ± 0.028b	1.06 ± 0.019b	1.06 ± 0.33b
Glycogen	0.16 ± 0.002a	0.16 ± 0.006a	0.12 ± 0.003b	0.12 ± 0.0005b
Triacylglyceride	0.22 ± 0.003a	0.21 ± 0.002a	0.13 ± 0.006b	0.097 ± 0.003c

At each row, different letters show statistical differences of values (Tukey's test, p ≤ 0.05).

## Discussion

Since chemical insecticides cause severe concerns regarding environment, non-target organism and biosafety of food, general trends have been directed to investigate on plants and microbes derived materials in pest control. Plants have genes that encode toxic proteins to overcome herbivores attacks. These toxic encoding genes have been considered as lectins, α-amylase inhibitors, protease inhibitors, and ribosome inactivating proteins (Fitches et al., 1997; de Oliveira et al., 2011; Saadati and Bandani, 2011). Among these molecules, lectins have shown significant effects on different life stages of many insects such as Coleoptera, Diptera, Hemiptera, Homoptera, Hymenoptera, Isoptera, and Lepidoptera (de Oliveira et al., 2011). In the current study, the effects of CCA added into artificial diet of *E. ceratoniae* larvae were determined on growth, survival, digestive enzymes and storage macromolecules. In the current study, CCA significantly affected survival, pupal weight and pre-adult period. Similar results have been obtained by other researchers using different lectins. Fitches et al. (1997) found that biomass of *Lacanobia oleracea* L. (Lepidoptera: Noctuidae) larvae decreased 32 and 23% after feeding on artificial diet containing 2% lectin and transgenic potato expressing 0.07 of total soluble protein of *Galanthus nivalis* lectin (Fitches et al., 1997). Also, it was revealed retarded larval development, pupal morphology and decreased survival below 40%. Expressed *Galanthis nivalis* lectin in transgenic rice plants showed resistance to *Nephotettix virescens* Distant (hemiptera: Cicadellidae) and *Nilaparvata lugens* Stal (Hemiptera: Delphacidae) by reduction of nymphal survival up to 50% (Foissac et al., 2000; Nagadhara et al., 2003). Gatehouse et al. (2007) reported the expression of GNA in transgenic potatoes for the control of the tomato moth, *L. oleracea* with a significant effect on larval size and adverse effect on the developmental rate (Gatehouse et al., 2007). Larval weight of *Anagastra kuehniella* Zeller (Lepidoptera: Pyralidae) decreased up to 84% after feeding on an artificial diet containing a lectin from *Koelreuteria paniculata* seeds (Macedo et al., 2003). Coelho et al. (2007) found that *Anona coriacea* lectin decreased larval weight of *A. kuehniella* approximately 50% when a concentration of 1% added into artificial diet. Macedo et al. (2003) assayed a leaf lectin from *Bauhinia monandra* against *A. kuehniella, Callosobruchus maculatus* Fabricius (Coleoptera: Bruchidae) and *Zabrotes subfasciatus* Boheman (Coleoptera: Bruchidae). It was found that a concentration of 1% in the artificial diet decreased larval weight to 40% while concentrations of 0.5 and 0.4% caused 20 and 50% reduction in the mass of *Z. subfasciatus* and *C. maculatus*, respectively. Sadeghi et al. (2006) demonstrated that feeding of larvae of *Spodoptera littoralis* Fabricius (Lepidoptera: Noctuidae) on tobacco leaves expressing *Allium porrum* lectin at 0.7 %, reduced the larval weight gain by 15–27%. Also, mortality of about 28% was observed in the intoxicated larvae. de Oliveira et al. (2011) demonstrated that cMoL showed a dose-dependent effect on average larval weight of *A. kuehniella*, a significant increase in total development time of 15 days and increased the rate of pupal mortality by 27.6%. These findings could be attributed to possible intervening of lectins in biological process of insects leading to longer development time and higher mortality. Meanwhile, feeding on the diet containing CCA caused lower feeding efficiency and smaller size of larvae and pupae.

Feeding on the diets containing different concentrations of CCA decreased digestive enzymatic activities in the treated larvae in comparison with control except for TAG-lipase. These results were proven by zymogram analysis for each enzyme. Higher concentrations of CCA decreased band sharpness and disappeared some isoenzymes of all assayed enzymes in comparison with control. Reduction of digestive enzyme activity could be explained by the complete study of Hamshou et al. (2010) on the effect of *S. sclerotiorum* agglutinin (SSA) on *A. pisum*. Histofluorescence studies on obtained sections from aphids fed on an artificial liquid diet containing FITC-labeled SSA indicated brush border zone of midgut as the primary target for SSA. In addition, exposure of insect midgut CF-203 cells to 25 mg/ml SSA resulted total loss of cell viability. Results provided strong evidence that SSA binds with specific carbohydrate moieties on the cell membrane proteins to start a signaling transduction cascade leading to death of the midgut epithelial cells, which causes insect mortality (Hamshou et al., 2010). Since, secretion of digestive enzymes was made from midgut cells via apocrine and holocrine process, midgut cell death decreased the amount of secreted enzymes by interrupting these processes. Ramzi and Sahragard (in press) found that CCA 2% significantly inhibited amylolytic activity of *E. ceratoniae* in mixed inhibition situation so that inhibition was dependent to pH and temperature. So, reduced activities of digestive enzymes could be due to a dual process; disruption of midgut cells and biochemically inhibition of the enzymes.

Macromolecules stored in fat bodies of insects have critical role to provide energy for biological processes due to metabolism of accumulated macromolecules like proteins, glycogen and triacylglyceride. In this study, significant reduction were found in amount of accumulated macromolecules. Moreover, it was previously indicated, different concentrations of CCA significantly decreased nutritional physiology of *E. ceratoniae*. In fact, feeding on CCA interrupted nutritional physiology resulting in less accumulated macromolecules in fat bodies. Also, indicated chronic effects of CCA depleted these storage molecules for detoxifying instead of common biological processes. These macromolecules were used in some situations like flight, starvation, feeding stresses and etc. In our case, lectin do intervene in routine digestion of food by the larvae and it put negative effects on nutrient digestion and absorption. Due to this anti-nutritive status, the larvae intend to utilize their stored macromolecules for biological processes.

In conclusion, extracted lectin from *C. colocynthis* significantly interrupted ecology and physiology of *E. ceratoniae* so further research and investigation seems to be necessary to closer attention in control programs of the pest as an alternative method. These interruptions reduce availability of nutrients especially carbohydrates and amino acids for larval growth leading to poor development. Hence, CCA possesses a great potential as a less harmful biotechnological tool which promotes an environment-friendly agriculture.

### Conflict of interest statement

The authors declare that the research was conducted in the absence of any commercial or financial relationships that could be construed as a potential conflict of interest.
